# Investigation of dust storms entering Western Iran using remotely sensed data and synoptic analysis

**DOI:** 10.1186/s40201-014-0124-4

**Published:** 2014-10-29

**Authors:** Ali D Boloorani, Seyed O Nabavi, Hosain A Bahrami, Fardin Mirzapour, Musa Kavosi, Esmail Abasi, Rasoul Azizi

**Affiliations:** Department of Remote Sensing & GIS & Geoinformatics Research Institute (GRI), University of Tehran, Tehran, Iran; Geoinformatics Research Institute (GRI), University of Tehran and Department of Geography and Regional Research, University of Vienna, Vienna, Austria; Department of Soil Science, Tarbiat Modares University, Tehran, Iran; Faculty of Electrical Engineering, Sadra Institute of Higher Education, Isfahan, Iran; Geoinformatics Research Institute (GRI), University of Tehran, Tehran, Iran

**Keywords:** Dust source, Dust storm, Dust detection, Remote sensing, Synoptic climatology

## Abstract

**Background:**

One of the natural phenomena which have had considerable impacts on various regions of the world, including Iran, is “dust storm”. In recent years, this phenomenon has taken on new dimensions in Iran and has changed from a local problem to a national issue. This study is an attempt to investigate the formation of the dust storms crossing the Western Iran.

**Methodology:**

To find the sources of the dust storms entering Iran, first we examine three determined dust paths in the region and their temporal activities, using MODIS satellite images. Then, four regions were identified as dust sources through soil, land cover and wind data. Finally, atmospheric analyses are implemented to find synoptic patterns inducing dust storms.

**Results and discussion:**

Source 1 has covered the region between the eastern banks of Euphrates and western banks of Tigris. Source 2 is in desert area of western and south-western Iraq. Finally source 3 is bounded in eastern and south-eastern deserts of Saudi Arabia called Rub-Al-Khali desert, or Empty Quarter. Moreover, south-eastern part of Iraq (source 4) was also determined as a secondary source which thickens the dust masses originating from the above mentioned sources. The study of synoptic circulations suggests that the dust storms originating from source 1 are formed due to the intense pressure gradient between the low-pressure system of Zagros and a high-pressure cell formed on Mediterranean Sea. The dust events in sources 2 and 3 are outcomes of the atmospheric circulations dominant in the cold period of the year in mid-latitudes.

## Background

**Figure 1:**
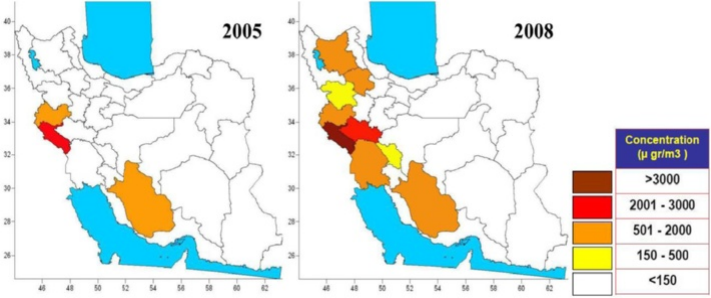
**Maximum concentration of dust particles (μg/m** ^**3**^ **) in affected provinces of Iran for a 3-year period 2005–2008 [** 3 **].**

Dust storms are the result of the air turbulences which spread a large mass of dust in the atmosphere, and decrease the horizontal visibility to less than 1000 meters [1]. In a general perspective, primary sources of dust plumes lie in the arid and semi-arid regions in East Asia, Middle East, Europe, Latin America, North America, Australia, eastern and southern Africa. Among them, Middle East has many dust sources in Arabian Peninsula, Syria, Egypt, Iraq and Iran [2]. Therefore, in countries with arid climate such as Iran, dust phenomena are not new natural events. However, in recent years there has been a new problem: a sudden increase in the number of dusty days and the level of air dust, especially in western Iran which has seriously affected the people’s health and everyday life. As shown in Figure 1, only in the course of 3 years (2005–2008), the dust event spotted in western Iran has considerably become wider in terms of thickness and spatial expansion.

The frequent occurrence of this phenomenon in various regions of the world such as Iran has gained attentions from academia and has triggered many research projects on the issue. Remote sensing is one of the most widely-used methods in dust storm studies. Drawing upon a model based on surface conditions and satellite observations, Ozsoy et al. examined one of the greatest dust events in Sahara Desert during the April of 1994 which affected a wide region from Caribbean to Eurasia [4]. El-Askary et al. argued that a synthetic approach is the best way of observing and tracking dust in satellite images [5]. Measuring solar reflectance bands, Qu et al. detected dust masses in satellite images [6]. Alles used air pollution indicatives and MODIS^a^ images to determine China’s dust sources; the results have shown that the phenomenon has originated in the cold regions and has caused many problems in Gobi desert while crossing and loading enormous dust particles in this region [7]. Li et al. have analysed the greatest dust in the eastern Australia using MODIS images and (BTD^b^) index. He detected the high performance of the method in identification of dust masses [8]. Climatological analysis, especially the synoptic approach - the study of relations between global atmospheric circulations and regional and local climates [9] - has been used in most of the researches performed on the atmospheric conditions during dust events. Investigating the transfer of dust from Sahara to Italy and central Europe, Barkan et al. recognized sub-tropical high pressure system on the south-eastern Mediterranean Sea and a low-pressure centre resulted from Iceland’s trough in the western Africa as the primary factors of dust formation and mobilization [10]. Zolfaghari and Abedzadeh adopted a synoptic approach to investigate the atmospheric systems leading to dust in western Iran in a 5-year period. They have concluded that Azur’s high-pressure system along with westerly-travelling cyclonic and anti-cyclonic systems are the most effective atmospheric factors on the dust events in the region [11]. Dayan et al. have classified dominant synoptic patterns in the region on dusty days and indicated that 60% of the dust events in the south-eastern Mediterranean occur as a result of Cyprus low-pressure system [12]. Gerivani et al. have adopted a different approach and categorized the sources of the dust storms in Iran based on geological maps and the information on wind erosion of the susceptible lands [13]. Although, all the mentioned studies have provided considerable results, the fact that a dust event results from multiple factors requires considering synthetic approaches. Utilizing various perspectives such as remote sensing data, synoptic climatology analysis, and ancillary data including soil texture and land cover of the studied regions, as well as the wind speed and direction maps, the present research tries to study the dust events in western Iran more precisely.

On the other hand, the most climatological studies of the dust storms in Iran are limited to a few numbers of case studies. Moreover, in these works the source-based classification of atmospheric circulations has not been considered to account for environmental conditions leading to dust storm. Therefore, in this work, we have increased the number of investigated events to 40 cases and also atmospheric aspects of dust storms are precisely studied. Significant rise in the number of recent dust storms in Iran and also applying new and up-to-date methods to examine this natural event provide another motivation for the present study.

The rest of this paper is organised as follows. Methods and data are presented in the next section. The research findings are discussed in section 3, and section 4 concludes the paper.

## Data and methods

### Data and methods for the identification of dust sources

**Figure 2:**
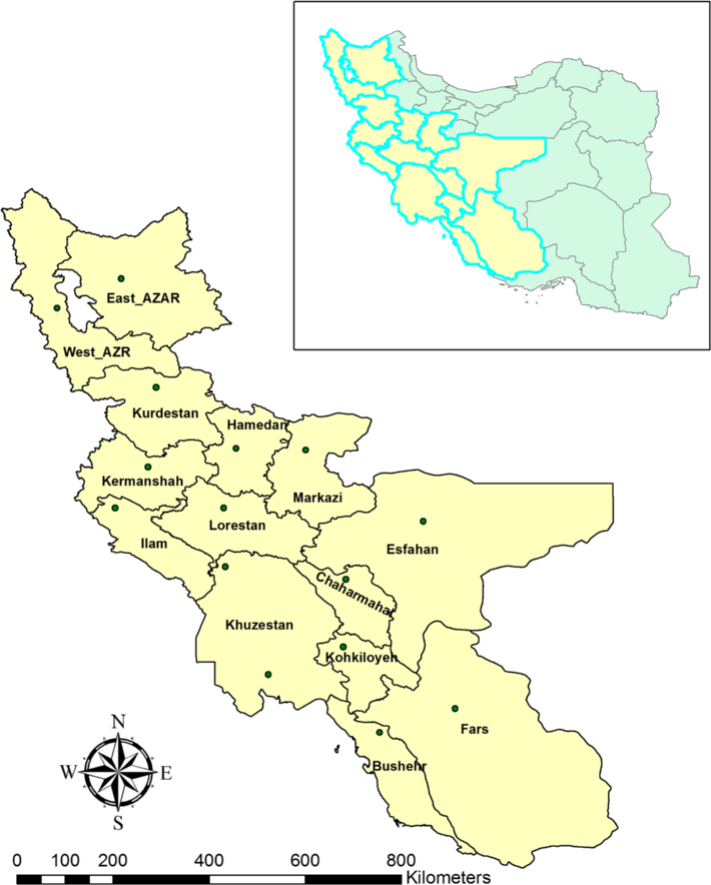
**Studied region and synoptic stations located in western Iran.**

To study the dust storms entering western Iran, daily data from 15 synoptic stations (see Figure 2) in a period from 2000 to 2008 are exploited.

In this research, only the dust events satisfying the following criteria were studied: (I) recorded at least in 3 synoptic stations, (II) rising from non-local origins, and (III) leading to a maximum horizontal visibility of 1000 meters.

In cases, the stations in the region under study indicated consecutive days with dust, only those days were specified as independent event when dust particles were injected to atmospheric currents from dust-prone areas. Whereas, maybe because of air stillness, dust continued for days over a region without any new dust injection, those dusty days were considered as on dust event.

Mobility of dust plumes from the originating sources to the surrounding regions do not necessarily follow density reduction regime, i.e., there may exist some other dust sources throughout the dust paths which inject new dust particles into the primary mass. Therefore, in the present study, the region where dust originates is not considered as the only source of the event. In fact, all the regions located in the dust path are determined using remote sensing imagery and then, supplementary data and information are taken into account to locate the dust sources more precisely. In order to identify dust paths and to estimate primary and secondary dust sources, approximately 100 MODIS images of Middle East were processed within a five-day period before recording time of each event.

In this research, image processing for dust detection is based on Ackerman’s method [14], arguing that brightness temperature difference of dust particles in bands 31 and 32 (11 and 12 micrometre wavelengths) is below zero in Kelvin scale. However, it is empirically considered −0.5 in the present work. To distinguish dust particles suspended in the air from land covers, two types of test were used; R-test and BTD-test [15]. After identifying possible dust sources and the relocation paths, more precise identification processes are performed by examining the soil data and land covers of the studied regions, and wind maps.

The following databases have provided the data used in the present study:

- Visibility data from Iranian Meteorology Organization database,
- MODIS images from NASA website,^c^
- Soil information from the Harmonized World Soil Database [16], and
- Land-cover data from ESA website^d^.

### Data and methods to identify atmospheric circulations associated with dust storms

The analysis of synoptic circulations associated with dust storms is carried out according to geopotential height at 850 hPa and 500 hPa levels, temperature, and wind data at height of 10 meters above the ground during the beginning days of studied dust storms. All atmospheric data, except for dust records, were provided by National Centre of Environmental Predicting (NCEP) database [17].

## Result and discussion

### Identification of western Iran’s dust sources

**Figure 3:**
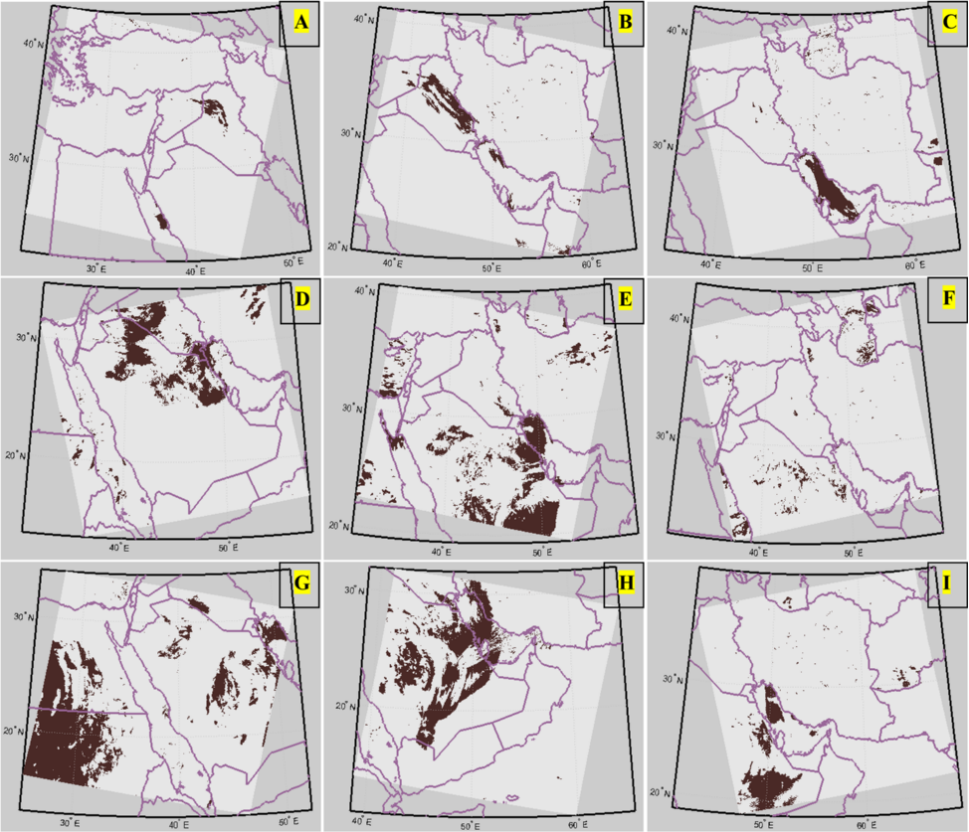
**Three specified paths of dust relocation to the western Iran.** Images in the first row are the samples of dust plumes moving along the first path (from left to right): the origin of primitive dust mass **(A)**, the dust mass relocating and entering to the study region **(B)**, and its dissipation **(C)** for August 7-8-9,2005. The images shown in the second and third rows show these three steps in the same order for dust events of February 15–16, 2004 **(D,** **E, and** **F)** and of March 1–3, 2007 **(G**, **H, and** **I)**.

Having examined the recorded dust storm data of the local stations from 2000 to 2008, a number of 40 dust events were selected. The analysis of the satellite images of dust events in the western Iran shows that the dust plumes travel in three main paths (from the source to the region under the study):

- *North western–South eastern path*: The detected dust plumes in Iraq and Syria are carried to the western part of Iran by north-westerly winds (Figure 3). Among the 40 dust storms, 23 events have formed along this path (Table 1).

**Table 1:** **The dates of the dust events which have formed and moved through the Northwest-Southeast path**

**No**	**Starting date of dust storm**	**Observation date of dust storm in study area**
1	11-Jun-05	12-Jun-05
2	12-Jun-05	13-Jun-05
3	22-Jun-05	23-Jun-05
4	24-Jun-05	25-Jun-05
5	04-Jul-05	05-Jul-05
6	24-Jul-05	25-Jul-05
7	07-Aug-05	08-Aug-05
8	08-Aug-05	09-Aug-05
9	17-Apr-06	18-Apr-06
10	17-Jul-07	18-Jul-07
11	18-Jul-07	19-Jul-07
12	15-Feb-08	15-Feb-08
13	04-Apr-08	05-Apr-08
14	29-Apr-08	01-May-08
15	01-Jun-08	02-Jun-08
16	07-Jun-08	08-Jun-08
17	08-Jun-08	09-Jun-08
18	15-Jun-08	16-Jun-08
19	16-Jun-08	17-Jun-08
20	17-Jun-08	18-Jun-08
21	18-Jun-08	19-Jun-08
22	30-Jun-08	02-Jul-08
23	15-Sep-08	16-Sep-08

Based on the recorded data, massive dust storms entering Iran began to move in this path since 2005 and hit a peak in 2008. Four consecutive dust events that have affected Iran since June 15 to 19, 2008 are rare instances of this phenomenon resulted from dust source activities in the first dust pathway. Therefore, it may be inferred that dust masses which have entered Iran in the recent years, have mainly moved along this path and are originated from the same sources. Seasonal distribution of dust events (except for an event in February 2008) indicates that this path is mainly active in the warm months of the year, especially June and July. The reasons for these circumstances will be discussed in details in chapter 3.2.

- *West-east path*: dust masses rise from Iraq-Jordan borders and enter Iran (Figure 3). 14 cases of the studied dust events have occurred along this path (Table 2).

**Table 2:** **The dates of the dust events which have formed and moved through the West–east path**

**No**	**Starting date of dust storm**	**Observation date of dust storm in study area**
1	21-Dec-02	22-Dec-02
2	22-May-03	22-May-03
3	15-Feb-04	16-Feb-04
4	13-May-04	14-May-04
5	15-May-04	16-May-04
6	06-Jan-05	07-Jan-05
7	24-Jan-05	25-Jan-05
8	03-Apr-05	04-Apr-05
9	05-May-05	05-May-05
10	21-Jan-06	22-Jan-06
11	04-Feb-07	04-Feb-07
12	16-May-07	17-May-07
13	19-Feb-08	20-Feb-08
14	15-Mar-08	15-Mar-08

Temporal distributions of the west–east dust events indicate a declining trend in their frequency by the end of the study period (in contrast to the dust events corresponding to the first path). According to Table 2, events of this category have mostly occurred in 2004 and 2005. These conditions again reflect the considerable effect of dust origins in recent dust events. The seasonal distribution of the dust storms formed in second path shows a peak in the cold period of year.

- *Southern-northern path*: It begins from the regions located to the south of Persian Gulf and ends at western Iran (Figure 3). Only three dust events travelled on this path (Table 3).

**Table 3:** **The dates of the dust events which have formed and displaced through the southern-northern path**

**No**	**Starting date of dust storm**	**Observation date of dust storm**
1	22-Mar-03	23-Mar-03
2	01-Mar-07	03-Mar-07
3	10-Apr-07	11-Apr-07

As indicated in Table 3, dust masses moving in the third path do not play a significant role in dust events occurring in Iran and thus, it cannot be considered as an influential region of forming the dust storms occurred in the last decade in the study area. Saudi Arabia dust events occurred in the late cold period of the year that is examined in atmospheric analysis section.

**Figure 4:**
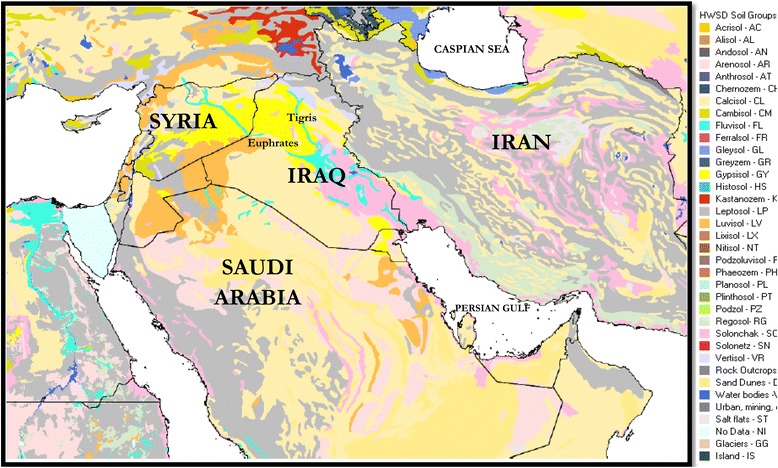
**The Middle East Soil Map [** 16 **].**

**Figure 5:**
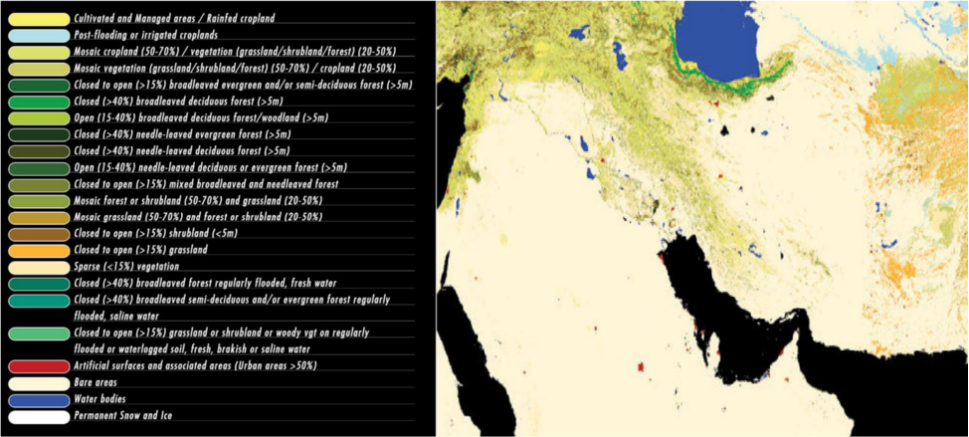
**Land-cover map of the region [** 18 **].**

**Figure 6:**
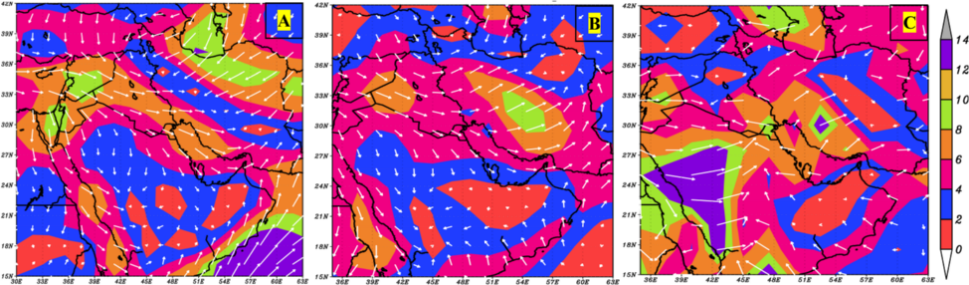
**Composite maps of the wind speed and direction at 10 meters above the ground for beginning days of dust storms originated along dust paths one (A), two (B) and three (C); the white arrows denote the wind direction and the background depicts the wind speed in m/s.**

Although it is possible to specify the approximate places of dust formation and its relocation paths by processing satellite images, the information on the soil and wind in the region should be analysed to identify the dust sources more accurately. Therefore, we carefully examined the ancillary data corresponding to the mentioned dust paths. These data include soil information of Iraq, Syria, and Saudi Arabia (Figure 4 and Table 4), their land covers data (Figure 5), as well as the maps of wind velocity and direction (Figure 6).

**Table 4:** **Soil characteristics of dust sources** [16]

**Sources**	**Dominant soil group**	**Topsoil USDA texture classification**	**AWC (mm)**	**Topsoil sand fraction (%)**
The east of Syria and North west of Iraq	GY - Gypsisols	loam	15-50	sand(35)/Silt(45)/clay(20)
South east of Iraq	Sc-Solonchaks	loam	150	sand(36)/Silt(43)/clay(21)
The west and south west of Iraq	CL-Calsisol	loamy sand	15-50	sand(39)/Silt(37)/clay(24)
The east and south east of Saudi Arabia	DS-sand dunes	loamy sand	15-100	sand(84)/Silt(9)/clay(7)

Soil maps of the northwest–southeast dust path in Syria and Iraq indicated a region of gypsum sediments in the north-western Iraq and the eastern Syria. This area has mainly formed in arid regions with poor vegetation called gypsisole (Figure 4). Despite the fact that southernmost region of the path, i.e. the south-eastern Iraq, consists of solonchaks soil, which is also found in arid and semi-arid regions, one of its distinguishing features is that the water table is close to the surface. Since, the soil is almost wet- a condition sometimes leading to water stillness on the soil surface [19]. Although in the recent years, this region has experienced an increase in depth of groundwater because of drought, and some ponds have changed into limited erodible zones [20], the south eastern Iraq cannot be considered as a main and wide dust source influencing the western Iran. However, in some cases, dust masses originating from other region have been intensified locally in this region and then entered Iran (not shown here). The quantitative characteristics of soil along northwest–southeast path (80% of the soil has a fine texture including clay and silt) verify that the north-western parts of Iraq and the eastern parts of Syria potentially lead to massive dust storms. Furthermore, these conditions are intensified by the regions with low soil moisture (15–50 mm per 1 m soil depth) and spare land covers. It should be noted that despite the fine texture of the soil in south eastern Iraq, higher moisture (150 mm per 1 m soil depth) and more dense land cover lead to a lower chance of dust event in this region (Table 4, Figures 4 and 5). According to the discussion above, the upper zone of Mesopotamia (Iraq-Syria border) is the most likely region to provide airborne particles for the dust storms in western Iran along the northwest-southeast path.

The study of the ancillary data on path two shows that the calcisols covers most parts of the west and south western Iraq (Figure 4). Calcisols is mainly formed in arid and semi-arid regions especially in foothills, and is known as an alluvial type of soil with fine texture, which makes land cover potentially erodible [19]. Table 4 gives data for an equal mixture of sand, clay, and silt in the west and south western Iraq. In this region, the amount of soil (dominant soil) moisture is about 50 mm per 1 m soil depth (even as low as 15 mm in some regions), which results in sparse vegetation (Figure 5).

The path three just start from southeast of Arabian Peninsula (the desert region of Rub-Al-Khali or Empty Quarter) to southern coasts of Persian Gulf. This part of Arabia is Regosol soil which mainly is composed of gravel and sand coverings (i.e. 84%) (Figure 4 and Table 4). Since the soil has a coarse texture, the number of dust storms reaching Iran throgh this pathway is much less than that from other dust sources (i.e. 3 out of 40 cases). However, the intense dryness (amount of soil moisture in some regions is around zero) and lack of vegetation (Figure 5) result in a large number of limited daily local sand storms in eastern Arabian Peninsula.

According to Figure 6, in the first day of all the studied dust events, wind speed and direction maps are in accordance with the estimated dust sources. During dusty days of the path one the speedy winds (6 to 12 m/s) skirted on Iraq and south-eastern Syria have the potential to make dust plumes in erosion-prone regions (Figure 6A). The composite map of wind speed and direction shows speedy air streams during the beginning days of dust storms in path two. As a result, the western and eastern parts of Iraq are prone to dust if other conditions are available. Along with the formation of dust in path three, two convergent wind currents come together in eastern Saudi Arabia. Surface westerly winds, most likely intensified by temperature differences between the western mountainous regions and the eastern desert area, converge with easterly currents. This circulation transports large amounts of dust masses from the eastern Arabia to upper atmospheric levels and higher latitudes. In fact, because of the coarse soil texture of Saudi Arabia deserts, only such special atmospheric circumstances can make massive dust storms enter regional circulations and subsequently the western Iran (Figure 6C).

**Figure 7:**
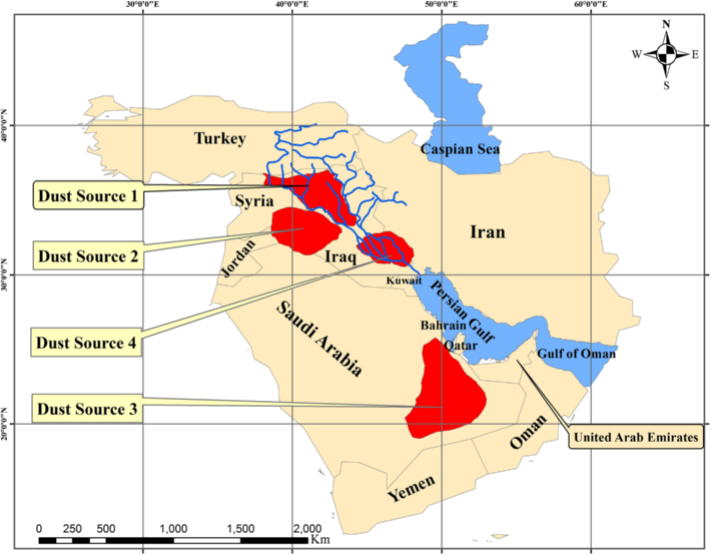
**Identified sources of dust storms from 2000 to 2008.**

Based on our findings the following regions are recognized as the main sources of dust events in the western Iran (Figure 7):

i. *Dust source 1: The region between eastern banks of Euphrates to western banks of Tigris*
  The region located in the north western Iraq and the eastern Syria was recognised as the main source of the dust storms entering western Iran. As discussed above, this region was the origin of 23 dust storms out of the 40. Most of dust events originating in this source have formed in Iraq and a few in Syria.
ii. *Dust source 2: The western and south western Iraq*
  14 cases out of 40 have formed in this region. It also includes parts of Jordan and Syrian Desert.
iii. *Dust source 3: The eastern and south-eastern Arabian Peninsula called Rub-Al Khali desert*
  Some parts of Rub-Al-Khali desert were the source of 3 cases out of the total 40.
iv. *Dust source 4: The south-eastern Iraq*
  This source is not considered as a major dust origin, but it intensifies the dust masses originating from other sources, especially from one and two dust formation areas. Therefore, the number of dust events formed in this region is not given separately.

Tracking dust storms and examination of various data and factors gave us most likely dust sources which send dust particles into Iran. However, the study of atmospheric conditions and identification of synoptic patterns during dust events is crucial to clarify predominant environmental conditions leading to dust storms.

### Atmospheric conditions and synoptic patterns

Before discussing the atmospheric conditions governing the occurrence of dust storms in the western Iran, it is necessary to note that the activity of each dust source follows certain temporal distribution. All the dust events originating in the source one, have occurred in the warm periods of the year (April to September), while other dust cases formed in the western and south western Iraq, and eastern and south eastern Saudi Arabia have been recorded in cold and late cold periods. The activity of the source four has happened in both, the warm and cold seasons. Such a clear distinction in the temporal distribution of the dust activities in the sources can be due to the formation of different atmospheric patterns in specific periods of the year. Synoptic circulations associated with the dust sources are discussed in the following subsections –except for atmospheric conditions affecting the fourth source, because it acts as the secondary origin coincidental to the primary sources.

#### Synoptic patterns of source one

**Figure 8:**
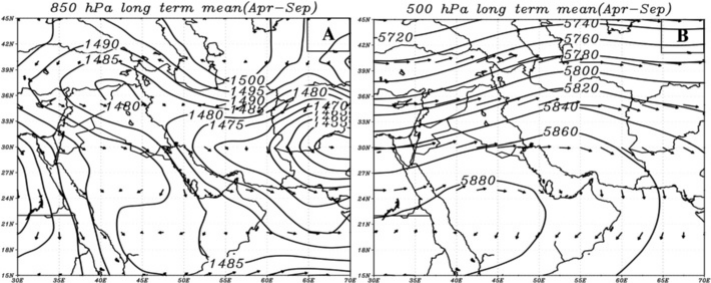
**Long-term mean of wind and geopotential height during warm times (Apr-Sep) at 850 hPa (A) and 500 hPa (B) levels.** Arrows and solid lines denote wind direction and geopotential height (in meters), respectively.

To identify synoptic circulations leading to dust events as extreme atmospheric patterns, their normal conditions should be examined first. As a dominant atmospheric circulation, a low-pressure centre is usually formed on Iraq during warm times. This low pressure system, formed in the lower troposphere, leads to winds blowing in northwest – southeast direction, known as Shamal Wind [21]. The increase in height causes the low-pressure tongue to expand onto southern regions of Turkey in north of Iran and toward Zagros Mountains in east (Figure 8A). Studies conducted on this circulation system gave the surface heating of Zagros Mountains as the main contributor in formation of this low [22,23]. Zagros low-pressure turns into a ridge and anti-cyclonic circulation in mid-troposphere (Figure 8B) strengthened by surface diabatic processes [23].

**Figure 9:**
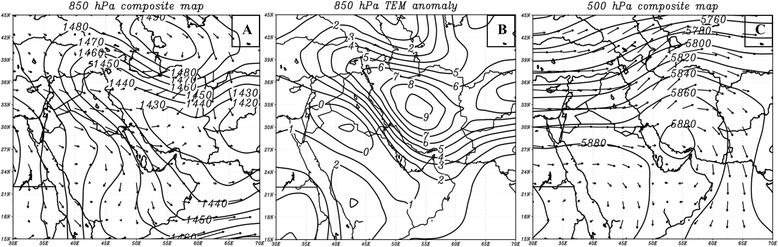
**Geopotential Height at 850 hPa composite map (A), temperature anomalies at 850 hPa (B) and Geopotential Height at 500 hPa composite map (C) during the formation of primitive dust masses of 23 events in source one (see Table** **1** **).**

The most striking feature of extreme synoptic patterns coincidental to most dust storms originating in source one is the expansion of Iraq low-pressure and its movement toward the western Iran and south of Turkey. This low encounters Azores’ high pressure formed on central Europe and northern banks of Mediterranean Sea at 850 hPa (Figure 9A). In 850 hPa composite map, large parts of Iran especially the western part, have experienced 1430 to 1440 geopotential meters. Whereas, in the long-term 850 hPa the geopotential heights 1475 to 1480 have been recorded for western Iran. These pressure values refer to a very warm and low centre that becomes dominant on the Iranian plateau simultaneous with dust storms in the study area. Another part of this circulation is the progression of Azores’ high pressure on Mediterranean Sea. This condition brings a high pressure gradient between these systems and, therefore, speedy winds through Syria and Iraq which causes very strong Shamal winds. These winds have the potential to raise dust plumes from surface to upper levels.

To explain how this circulation pattern is formed, the effects of seasonal thermal processes should be examined. In summertime, apart from the low pressure of the Bay of Bengal, another thermal low forms in Pakistan and sometimes in the north-western India known as Paki-India low. According to Bollasina and Nigam, the intensified surface heating of the Iranian plateau, especially Zagros Mountains, is one of the factors involved in deepening Paki-India low and its movement into Iran [24]. As mentioned before, surface heating of Zagros Mountains in normal conditions also leads to a low-pressure centre at 800–850 hPa levels. But along with Iran dust storms, heating process of Zagros Mountains and eastern deserts of Iran increases dramatically (Figure 9B). Based on the classic definition of thermal atmospheric circulations, this condition also results in the formation of high and low pressure systems in upper and lower-troposphere, respectively. Records of temperature in the composite map at 850 hPa show anomalies^e^ over 6 to 9 degrees Celsius in Zagros Mountains. 500 hPa composite map for dusty days occurred in North West Iraq and east Syria origins shows relocation of tropical high air mass, indicated by 5880-gpm contour, to the southern Iran which locates over Saudi Arabia Peninsula in the long term map (Figure 9C). Given that 500 hPa level is not usually affected by low surface thermal conditions, the locating of this high could be due to the significant rise in surface temperature on Zagrus Mountains. It is necessary to note that the deepening of low-pressure system located on Iraq and western Iran is not only affected by surface diabatic heating but also in some cases it is affected by dynamic processes resulted from travelling troughs [9]. Within the beginning days of dust storms formed in source one, trough patterns, located on Iraq and subsequently Iran, might deepen lower-troposphere thermal cyclones.

#### Synoptic circulations during dust events originating from sources 2 and 3

**Figure 10:**
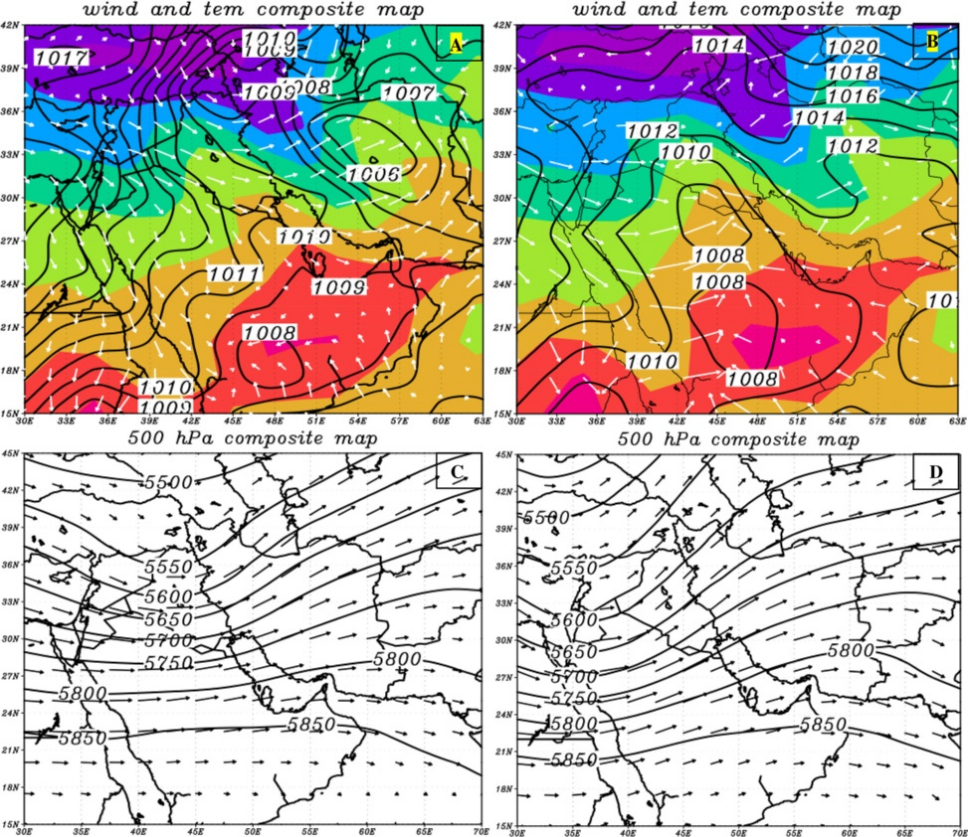
**Composite maps of sea-level pressure (A) and 500 hPa geopotential height (C) along with western and south-western Iraq dust events along with dust source two; composite maps of sea-level pressure (B) and 500 hPa geopotential heights (D) for source three.** Coloured background in figures **(A)** and **(B)** denotes surface temperature in Celsius; solid lines indicate surface pressure in hPa; arrows depict wind direction.

Since the temporal distributions of dust activities of the sources happen during cold months of year (October to March), it is expected that synoptic patterns governing these two sources to be similar. The analysis of atmospheric circulations of dust in the western and south western Iraq shows cyclonic and anti-cyclonic circulations as dusty patterns in the region. Almost all dust cases from western and south western Iraq begin when this source is put between travelling cyclonic and anti-cyclonic circulations. In other words, the cold section of cyclones brings high speed and dusty winds. This part of cyclonic circulation can be seen in average temperature for dusty days (Figure 10A). However, a few dust events were formed ahead of cold front or warm section in this region (not given here). Conditions have been partly different during the occurrence of dust events in eastern and south eastern Saudi Arabia. In these cases, dust masses are formed only in warm section of cyclonic circulations (Figure 10B).

The dynamic processes of westerly winds in mid-troposphere have a very significant role in the formation of surface mid-latitude cyclones and anticyclones. As seen in Figure 9C and 9D, cyclonic and anti-cyclonic circulations are formed respectively under the front and back of trough. During dusty days of the western and south western Iraq, a trough lies at 500 hPa level which leads to the formation of cyclonic (low pressure) and anti-cyclonic (high pressure) circulations in Iran and eastern shores of the Mediterranean sea. In fact, the dust source is strengthened by being located between low and high-pressure centres and subsequently, being affected through pre-cold front (cold sector), west–east, high-speed winds. While trough gets deeper, cyclonic circulation relocates toward lower latitudes on Saudi Arabia. In these cases, speedy winds causing extensive dust storms will form in warm sector of the cyclone with a roughly south–north direction.

## Conclusion

The present research uses multiple data and methods to determine the origin of the dust storms affecting the western Iran. By analysing the corresponding satellite images, three main paths of dust paths were recognized:

- *Path 1*: the northwest-southeast path, from the north-western Iraq and the south eastern Syria, to western Iran,
- *Path 2*: the second pathway with eastern-western direction, from Iraq-Jordan borders extended to the western Iran,
- *Path 3*: the last path from southern banks of Persian Gulf to western Iran through a south–north directing.

The role of the first path in the mobility of dust particles to the western Iran is much more significant than other two paths. The temporal distribution of western Iran dust events shows that the first dust path and its dust source have been abruptly activated from 2005 and hit a peak in 2008. Therefore, this path and its dust-prone regions can be considered as the main origin of recent dust storms entering western Iran. Dust events in paths one and two do not show significant rise in recent years. Dust events relocated from northwest-southeast path mainly occur in the warm periods of the year. In contrast, the paths of two and three have maximum seasonal activities in the cold months. The present research has used soil information, the type of land covers, and wind velocity and direction of sea level pressure to locate the dust sources more precisely. Analysing theses information, we found four main dust-prone regions which affect western Iran:

- *Source 1:* the eastern banks of Euphrates and the western banks of Tigris as main western Iran dust sources,
- *Source 2:* the western and south western Iraq, and also small parts of Syria and Jordan,
- *Source 3:* the eastern and south-eastern deserts of Arabia called Rub-Al-Khali Desert,
- *Source 4:* the south-eastern Iraq, as a secondary source.

The examination of the atmospheric conditions governing each dust source indicates that synoptic patterns during dust events originating from source one have been mainly affected by the surface diabatic processes. Whereas, dust storm in sources two and three are caused by cyclonic and anti-cyclonic circulations and are largely outcome of dynamic processes of westerly winds.

## Endnotes

^a^Moderate Resolution Imaging Spectroradiometer.

^b^Brightness Temperature Difference.

^c^http://rapidfire.sci.gsfc.nasa.gov/realtime.

^d^http://www.esa.int/Our_Activities/Observing_the_Earth/Space_for_our_climate/ESA_global_land_cover_map_available_online.

^e^Temperature anomaly = (daily temperature – average daily temperature).
